# Encapsulating peritoneal sclerosis in a patient after allogeneic hematopoietic stem cell transplantation: a case report

**DOI:** 10.1186/s12876-019-0933-0

**Published:** 2019-01-21

**Authors:** Yoshimitsu Shimomura, Shinsuke Sakai, Hiroyuki Ueda, Kohei Fujikura, Yukihiro Imai, Takayuki Ishikawa

**Affiliations:** 1 Department of Hematology, Kobe City Hospital Organization, Kobe City Medical Center General Hospital, 2-1-1 Minatojima-Minamimachi, Chuo-ku, Kobe, 650-0047 Japan; 2 Department of Nephrology, Osaka University Graduate School of Medicine, Osaka, Japan; 3 Department of Diagnostic Radiology, Kobe City Hospital Organization, Kobe City Medical Center General Hospital, Kobe, Japan; 4 Department of Clinical Pathology, Kobe City Hospital Organization, Kobe City Medical Center General Hospital, Kobe, Japan

**Keywords:** Allogeneic hematopoietic stem cell transplantation, Chronic graft-versus-host disease, Serositis, And encapsulating peritoneal sclerosis

## Abstract

**Background:**

Encapsulating peritoneal sclerosis (EPS) is a chronic clinical syndrome of acute or subacute gastrointestinal obstruction seen mainly in patients undergoing peritoneal dialysis. Although there are a few reports on EPS developing in non-peritoneal dialysis patients, it has not been reported in patients undergoing allogeneic haematopoietic stem cell transplantation (HSCT). Here, we report a case of EPS after a second HSCT.

**Case presentation:**

A 46-year-old man with myelodysplastic syndrome showed relapse after HSCT and received a second HSCT. The patient was diagnosed with chronic graft-versus-host disease (cGVHD)-associated serositis because of persistent ascites. His ascites improved gradually and disappeared without immunosuppressive therapy. He presented with nausea, weight loss, and constipation 1 year after improvement of ascites. Computed tomography revealed no organic obstruction, but did reveal dilated, thickened, and adhered small bowel loops with a mass-like appearance. He was diagnosed with EPS on the basis of clinical symptoms and image findings. He received corticosteroid therapy (20 mg/body) without any improvement in symptoms. He developed recurrence of myelodysplastic syndrome at 1 month after initiation of corticosteroid therapy. This progressed into acute myeloid leukaemia after 3 months. He died 31 months after the second HSCT. At autopsy, the small and large intestines had formed extensive adhesions and showed signs of progressive fibrosis with peritoneal sclerosis, fibroblast swelling, fibrin deposition, and inflammatory cell infiltration, which confirmed the diagnosis of EPS.

**Conclusion:**

This case suggests that EPS may complicate patients with cGVHD-associated serositis. Although the mechanism of EPS development is not clear, clinicians should be aware of this eventuality.

## Background

Allogeneic haematopoietic stem cell transplantation (HSCT) is a therapeutic modality to cure haematopoietic malignancies. Approximately 50% of the recipients develop chronic graft-versus-host disease (cGVHD) that can cause dysfunction of multiple organs and lead to morbidity and mortality [1]. Serositis is inflammation of the linings of the heart, lung, and abdominal organs, and causes pericardial effusion, pleural effusion, and ascites, respectively. Serositis can be caused by many different factors and is recognized as one of the rarest forms of cGVHD following allogeneic HSCT [2].

Encapsulating peritoneal sclerosis (EPS) is a chronic clinical syndrome of acute or sub-acute gastrointestinal obstruction seen mainly in patients undergoing peritoneal dialysis (PD). The cause of EPS is unclear, but it appears to develop through multiple factors such as chronic exposure to dialysate and chronic inflammation of the peritoneum [3]. Although there are a few reports on EPS developing in non-PD patients, it has not been reported in patients undergoing HSCT [4]. Here, we report the first individual who developed EPS after allogeneic HSCT.

## Case report

A 46-year-old man was admitted to our hospital because of fatigue and fever. On admission, a complete blood count revealed the following: haemoglobin 7.9 g/dL, platelet count 21 × 10^9^/L, and white blood cell count 0.4 × 10^9^/L. A bone marrow sample was markedly hypocellular, containing 1.4% myeloblasts and micromegakaryocytes. He was diagnosed with myelodysplastic syndrome (MDS) on the basis of morphological features. Chromosomal examination showed 42–44, XY, − 4, − 5, del (7), − 9, − 17, − 20, + 1-3mar, inc [6/6].

The patient received allogeneic HSCT using peripheral blood from a human leukocyte antigen (HLA) 6/6 matched sibling 1 month after hospitalization, with a conditioning regimen of cyclophosphamide (120 mg/kg) and total body irradiation (12 Gy). Neutrophil engraftment was achieved 21 days after the first HSCT. However, recurrence of MDS was confirmed 91 days after HSCT.

Four months after the initial HSCT, he received a second HSCT using HLA 4/6 bidirectional mismatched single unit cord blood with a conditioning regimen of fludarabine (150 mg/m^2^), melphalan (80 mg/m^2^), and busulfan (12.8 mg/kg). GVHD prophylaxis consisted of tacrolimus and mycophenolate mofetil. He achieved neutrophil engraftment at 15 days after the second HSCT. Immunosuppressive therapy was rapidly tapered and discontinued at 3 months after the second HSCT to prevent disease recurrence.

**Fig. 1:**
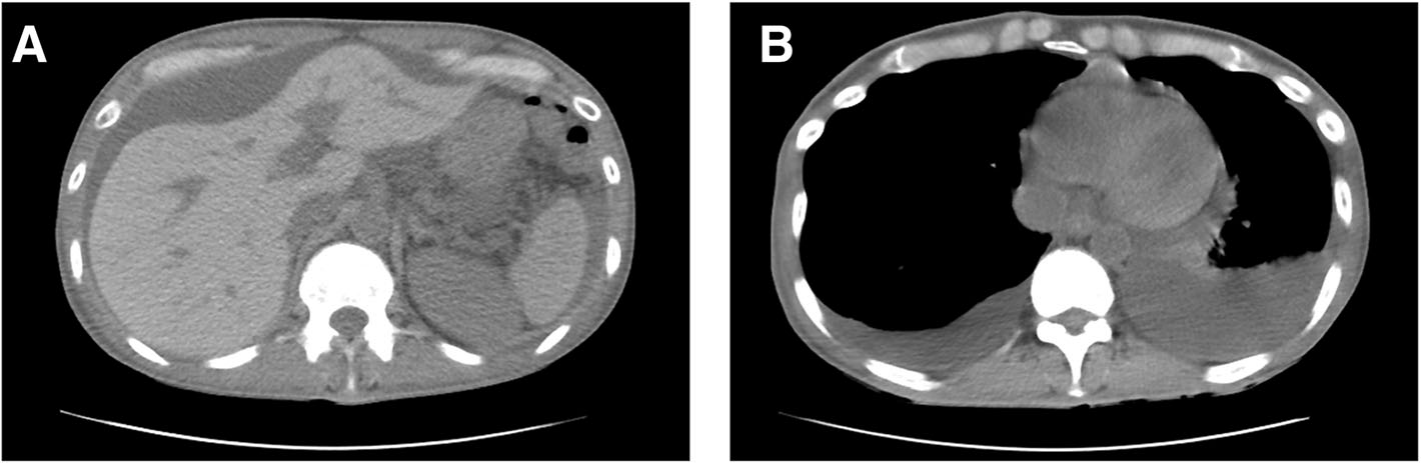
Computed tomography findings. Computed tomography findings at the time of development of persistent ascites (**a**) and pleural effusion (**b**)

The patient developed persistent ascites and pleural effusion 25 days after the second HSCT (Fig. 1). Initially, this was thought to be due to the engraftment syndrome because he presented with persistent fever without liver and kidney dysfunction. No additional immunosuppressive treatment was administered because he had no organ dysfunctions and had a high risk of disease. His symptoms, other than ascites and pleural effusion, were improved after observation. Ten months after the second HSCT, ascites inspection was performed because ascites and pleural effusion were sustained since the symptoms appeared. Ascites involved a yellow and exudative fluid with a serum ascites albumin gradient of 0.9. The fluid contained no abnormal cells. Staining and culturing for bacteria, fungi, and mycobacteria were negative. During that time, cough and dyspnea developed, and he was diagnosed with bronchiolitis obliterans because of decreased forced expiratory volume in 1 s and no findings on lung computed tomography. He was treated with clarithromycin and inhaled steroids but without a systemic corticosteroid. His serositis was also considered to be a cGVHD-related condition. His ascites decreased gradually and disappeared at 1 year after the second HSCT without a systemic corticosteroid or other immunosuppressive agents.

**Fig. 2:**
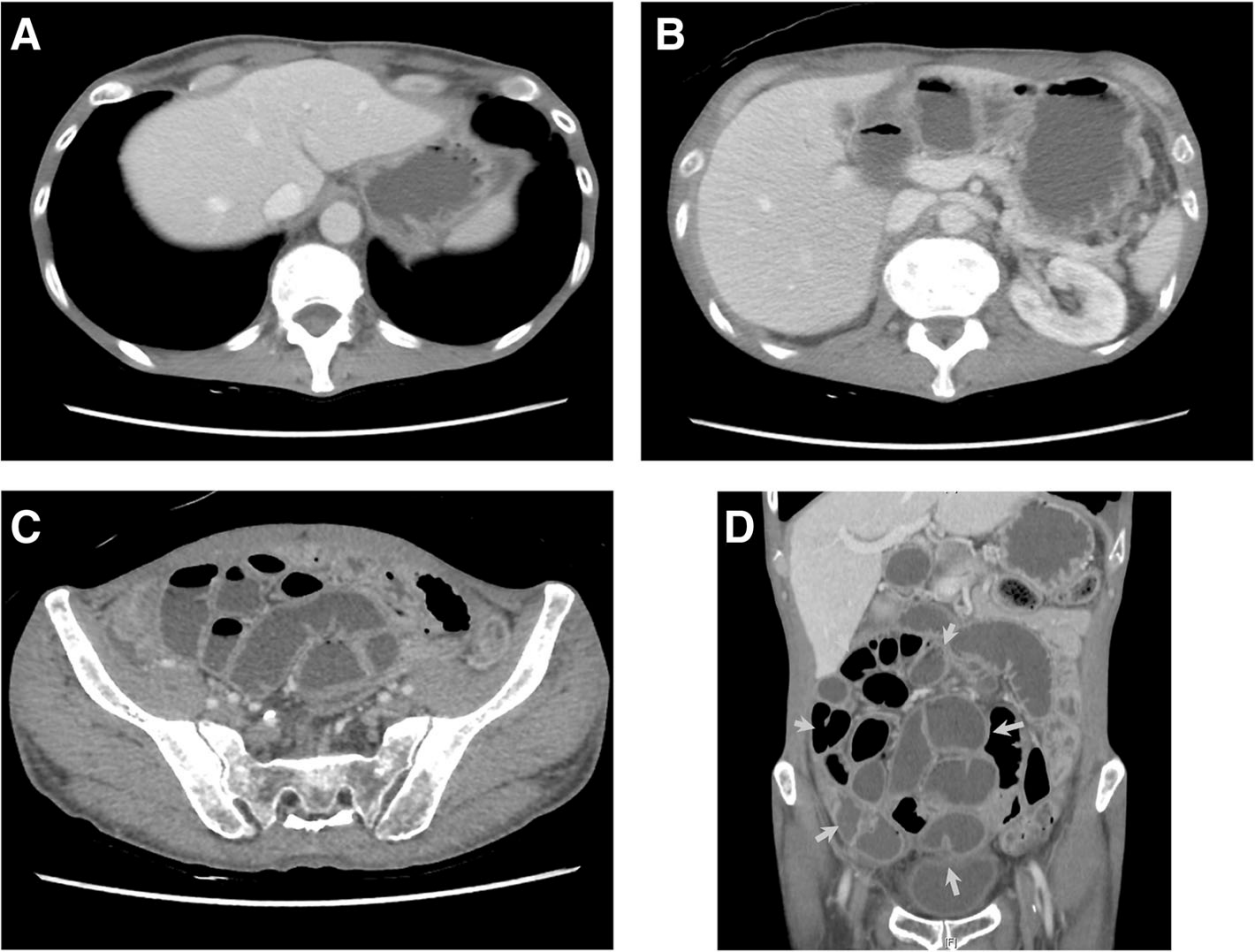
Computed tomography findings at the time of diagnosis of encapsulating peritoneal sclerosis. The patient had neither ascites nor pleural effusion (**a**, **b**). Computed tomography of the abdomen reveals dilated, thickened, adhered, and fluid-filled small bowel loops (**c**). A coronal reformatted computed tomography image shows that small bowel loops gathered to form a large mass as if they had a capsule (yellow arrows in **d**)

Two years after the second HSCT, he presented with nausea, weight loss, and constipation. Computed tomography revealed no intestinal obstruction, but did reveal dilated, thickened, and adhered small bowel loops showing a mass-like appearance as if they had a capsule (Fig. 2). Although peritoneum biopsy was not performed because of the concern about intestinal perforation (worsening symptoms), he was diagnosed with EPS on the basis of clinical findings. He received corticosteroid therapy (20 mg/body) without any improvement in symptoms. He developed recurrence of MDS at 1 month after initiation of corticosteroid therapy. This progressed into acute myeloid leukaemia after 3 months. He died 31 months after the second HSCT.

**Fig. 3:**
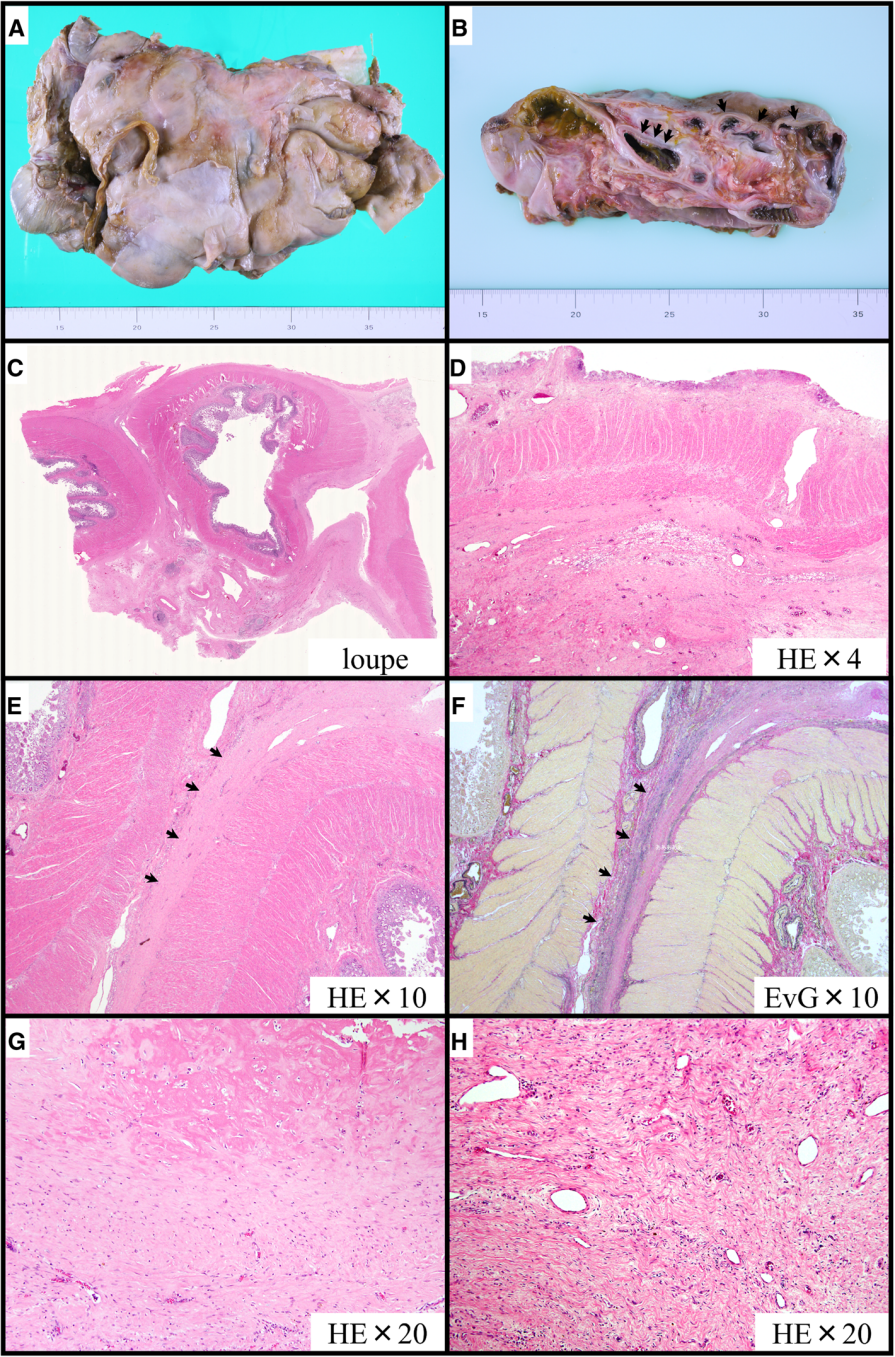
Pathological findings of encapsulating peritoneal sclerosis evaluated at autopsy. The macroscopic appearance reveals a cocoon-like encapsulation of the entire intestine (**a**). A macroscopic section of encapsulating peritoneal sclerosis shows bowel loops (arrows) covered by a thickened and fibrotic peritoneal membrane (**b**). Microscopic examination reveals marked subserosal fibrosis (**c**, **d**), adhesion of the serous membrane (**e**, **f** arrows), fibrin deposition (**g**), and fibroblast swelling with mononuclear cell infiltration (**h**). EvG; Elastica van Gieson, HE; Haematoxylin and Eosin

The patient’s family gave permission for an immediate autopsy of all organs to obtain a definitive diagnosis. Macroscopically, the entire intestine had adhered into a single “cocoon” with fibrotic peritoneal thickening (Fig. 3a, b). Histologically, intestinal loops were adhered to each other with intervening fibrosis, and showed signs of superficial fibrin deposition, fibroblast proliferation, and sclerotic fibrous peritoneal thickening with sparse inflammatory cell infiltration (Fig. 3c-h), confirming the most characteristic features of EPS.

## Discussion and conclusion

The current report describes a patient with EPS after allogeneic HSCT. He had persistent ascites and pleural effusion for a prolonged period after allogeneic HSCT. He exhibited severe cGVHD with lung involvement presenting as bronchiolitis obliterans. EPS-like symptoms developed after more than 1 year of gradually improving persistent ascites and developing severe cGVHD. His diagnosis was confirmed by autopsy. Although the cause of EPS is only speculative, this disorder may have been associated with cGVHD.

Serositis is recognized as inflammation of any of the serosal linings of the body. In a non-transplant setting, serosal inflammation can be caused by a variety of factors including infection, autoimmune disease, and malignant disease [2]. In the setting of HSCT, serositis is a well-recognized but rare manifestation of cGVHD-associated conditions [5]. Evidence regarding serositis after allogeneic HSCT is limited to case reports and a small case series because of its low frequency, with an incidence of 1–2% [2, 6, 7]. Seber et al. reported an association between unexplained effusions and acute and cGVHD [6]. Especially, the association with cGVHD was demonstrated because pericardial effusion or ascites developed late after allogeneic HSCT [7, 8]. Regarding treatment and outcomes, immunosuppressive therapy, such as corticosteroids, was reported to be effective and the prognosis was good with a median survival of 105 months [2, 7–9].

EPS is a fatal and rare complication occurring in patients undergoing long-term PD. The incidence of EPS was 0.7–3.3% in PD patients, and increased as the duration of PD was extended [10]. The exact aetiology of EPS in non-PD patients was unclear, but some risk factors were reported. Chronic inflammation and its associated ascites retention may also cause EPS in non-PD patients; for example, tuberculous peritonitis and autoimmune disease were reported [4].

Although the pathophysiology of EPS is also not fully understood, a two-hit theory is generally accepted [3]. The first hit causes mesothelial disruption that can trigger a fibrotic process. This usually occurs as a result of chronic exposure to dialysate in patients undergoing PD. The second hit, such as inflammation of the peritoneum and fibrin deposition, triggers the development of EPS [11, 12]. In addition, recent data suggested that an inflamed peritoneal membrane during PD was involved in the subsequent development of EPS by inducing fibrosis in PD patients [13, 14].

The prognosis of EPS is poor. Immunosuppressive therapy and surgery are the treatments of choice. Corticosteroid therapy is effective in the early stage of EPS, but the clinical response is reduced at later stages of the disease in patients who present with absolute bowel obstruction. The improvement of clinical symptoms in patients treated with corticosteroids is only 38.5% [15]. Although surgery can be performed in later stages of EPS and is effective, with a recovery rate of 58.3%, the recurrence rate and mortality are high [15, 16]. Thus, prevention of EPS is required.

In the present patient, EPS appeared to develop because of long-term ascites retention and chronic inflammation of the peritoneum caused by cGVHD-associated serositis. Probably, the first hit (mesothelial disruption) occurred as a result of cGVHD-associated peritoneal inflammation, and the chronic persistent inflammation in the peritoneal membrane induced fibrosis. The second hit might be chronic exposure to ascites. The patient had no risk factors of EPS, such as PD, intraperitoneal infection, or administration of drugs, and the timing of the onset of EPS was much faster than that reported previously [17]. This suggested that chronic inflammation caused by cGVHD may be associated with the development of EPS.

In conclusion, our experience demonstrated that EPS can develop in patients with cGVHD-associated serositis. Although the mechanism of EPS development is not clear, clinicians should be alert to this eventuality. Early therapeutic approaches to pleural, peritoneal, or pericardial effusion may be necessary to prevent the development of EPS.

## Abbreviations

cGVHD: chronic graft-versus-host disease
EPS: encapsulating peritoneal sclerosis
EvG: Elastica van Gieson
HE: Haematoxylin and Eosin
HLA: human leukocyte antigen
HSCT: hematopoietic stem cell transplantation
MDS: myelodysplastic syndrome
PD: peritoneal dialysis
